# Acupuncture Therapies for Chemotherapy-Induced Nausea and Vomiting in Patients With Breast Cancer: Protocol for a Systematic Review and Network Meta-Analysis

**DOI:** 10.2196/86384

**Published:** 2026-04-21

**Authors:** Yutao Luo, Zhujun Bian, Qi Wang, Tiantian Lei, Hong Zhao

**Affiliations:** 1 The First School of Clinical Medicine, Zhejiang Chinese Medical University, No. 548 Binwen Road, Binjiang District, Hangzhou, Zhejiang, China, 86 13588104817; 2 Department of Breast Surgery, Jiaxing Hospital of Traditional Chinese Medicine, Jiaxing, Zhejiang, China; 3 Department of Breast Surgery, The First Affiliated Hospital of Zhejiang Chinese Medical University (Zhejiang Provincial Hospital of Chinese Medicine), Hangzhou, China

**Keywords:** acupuncture, chemotherapy-induced nausea and vomiting, breast cancer, network meta-analysis, protocol

## Abstract

**Background:**

As a prevalent side effect, chemotherapy-induced nausea and vomiting (CINV) imposes a burden on the daily lives of patients with breast cancer. Multiple clinical trials have suggested the validity of acupuncture in alleviating CINV; however, the optimal acupuncture modality remains unclear.

**Objective:**

This protocol describes a systematic review and network meta-analysis to investigate the efficacy and safety of distinct acupuncture interventions for treating CINV in patients with breast cancer.

**Methods:**

Eight databases (PubMed, Embase, Web of Science, the Cochrane Library, China National Knowledge Infrastructure, VIP Database, Wanfang Database, and the Chinese Biomedical Literature Database) will be searched for eligible studies from their respective inception to July 31, 2025. The language of published studies is limited to English and Chinese. Primary outcomes are CINV intensity and clinical effectiveness rates. Secondary outcomes include recurrence rates, safety outcomes, and quality of life. Risk of bias will be assessed using the Cochrane risk of bias tool. Pairwise meta-analysis will be conducted in Stata using random-effects models with Hartung-Knapp-Sidik-Jonkman CIs. Network meta-analysis will be conducted using Bayesian Markov chain Monte Carlo methods in R software. Convergence will be assessed using Gelman-Rubin statistics and trace plots. Heterogeneity will be summarized using τ^2^ and τ, along with prediction intervals, when applicable. Consistency between direct and indirect evidence will be evaluated using the node-splitting method and design-by-treatment interaction test. Small-study effects will be assessed via comparison-adjusted funnel plots and the Egger test in Stata. Where feasible, subgroup analyses and meta-regression analyses will be performed. The certainty of evidence will be evaluated using the Grading of Recommendations Assessment, Development, and Evaluation framework.

**Results:**

A preliminary scoping search was completed in August 2025, and this protocol was finalized in October 2025. The comprehensive literature search and study selection are expected to be completed by June 2026, followed by data extraction by August 2026. Data synthesis and final manuscript preparation are scheduled to be completed by December 2026.

**Conclusions:**

This analysis will expand the range of evidence-based acupuncture options available to clinicians for treating CINV in patients with breast cancer.

## Introduction

Breast cancer is the most widespread malignant tumor in women and a major contributor to cancer-related deaths in female patients worldwide [1,2]. In the past few decades, the prevalence of breast cancer has risen dramatically, increasing the burden of the disease and making it a key public health problem worldwide. According to the Global Cancer Observatory statistics, the standardized incidence and mortality rates of female patients with breast carcinoma worldwide in 2022 were 47.8 per 100,000 and 13.6 per 100,000, respectively. Breast cancer leads globally in both incidence and fatality across most countries [2]. Currently, surgery, chemotherapy, targeted therapy, endocrine therapy, and radiotherapy are the leading treatments for breast cancer [3]. Chemotherapy can effectively prolong the life of patients [4] and plays an essential role in preoperative neoadjuvant therapy, postoperative adjuvant therapy, and advanced-stage treatment.

However, chemotherapy also causes a series of side effects, such as myelosuppression, cancer-related fatigue, nausea and vomiting, and liver and kidney toxicity [5]. Among these, chemotherapy-induced nausea and vomiting (CINV) remains a frequent adverse effect, occurring in about 70% to 80% of patients undergoing chemotherapy [6] and causing metabolic disorders and malnutrition. It greatly affects the patients’ daily lives and may even lead to fear of chemotherapy and a decline in compliance.

The mechanisms by which chemotherapy drugs cause nausea and vomiting are complex and not completely defined. It is generally accepted that most chemotherapeutic drugs irritate the mucosa of the gastrointestinal tract, causing mucosal damage and leading to the release of 5-hydroxytryptamine (5-HT) from enterochromaffin cells in the mucosa between the stomach and the ileum, which binds to appropriate receptors to generate nerve impulses that stimulate the vomiting centers, leading to the occurrence of vomiting [7]. In contrast, the chemoreceptor trigger zone, located at the base of the fourth ventricle of the brain, has a weak blood-brain barrier and is therefore susceptible to stimulation by chemotherapy drugs and their metabolites in the blood. In addition, substance P, which stimulates vomiting by binding to the neurokinin-1 (NK-1) receptor, is a critical neurotransmitter that causes CINV [8].

Antiemetic drugs in clinical use can be broadly categorized into 5-HT3 receptor antagonists (RAs), NK-1 RAs, glucocorticoids, phenothiazines, and others. Among these, 5-HT3 RAs are the primary medications used for the treatment of CINV in clinical practice, including ondansetron, granisetron, dolasetron, and tropisetron [9]. Unlike other 5-HT3 RAs, palonosetron is a safer and more efficient second-generation 5-HT3 RA [10]. It has an extended half-life of 40 hours and a significantly higher receptor affinity. It is especially useful for the prevention and mitigation of acute CINV. In contrast, NK-1 RAs and substance P are closely associated with delayed CINV [11]. Representative drugs include aprepitant, rolapitant, and netupitant, which have a broader range of antiemetic effects and fewer adverse effects. In addition, dexamethasone is often used in combination with other drugs in CINV treatment. Although there is no unanimous consensus on its antiemetic mechanism, it has been clinically shown that a combination of dexamethasone with RAs significantly relieves nausea and vomiting in patients undergoing chemotherapy [12]. Furthermore, for chemotherapy agents with different emetic risk classes, various combination regimens are formulated [13].

Although there is no concept of chemotherapy in traditional Chinese medicine (TCM), TCM possesses extensive and well-established therapeutic experience in effectively addressing nausea and vomiting. According to TCM theory, the pathogenesis of these symptoms is closely linked to the spleen, stomach, and *qi* (the fundamental substance that constitutes the body and sustains vital activities). The spleen-stomach system serves as the origin of *qi* and blood and is responsible for transforming food and transporting nutrients to nourish all *zang-fu* organs and body tissues. Pathogenic factors disrupt normal physiological functions, while chemotherapy-induced cytotoxicity damages vital *qi*. This leads to spleen-stomach deficiency, impaired transformation-transportation functions, dysregulation of *qi* ascending-descending movements, and stomach-*qi* ascending, which ultimately manifests as nausea and vomiting [14].

TCM explores various therapeutic approaches for nausea and vomiting, broadly categorized into internal and external therapies based on administration routes. Internal therapies primarily involve oral herbal decoctions, while external modalities focus on acupuncture and moxibustion. Compared to Western pharmaceuticals, TCM demonstrates advantages such as cost-effectiveness, minimal adverse effects, and procedural simplicity, making it widely adopted in CINV management globally [15-19].

Acupuncture achieves therapeutic effects through acupoint stimulation; therefore, the broader concept of acupuncture encompasses multiple stimulation techniques, including but not limited to electroacupuncture, auricular acupressure, fire needling, catgut embedding, intradermal needling, abdominal acupuncture, warm needling, conventional acupuncture, mild moxibustion, ginger-partitioned moxibustion, and acupressure. In clinical practice, practitioners may use single modalities or combinations of 2 to 3 techniques.

Although these interventions are extensively used clinically and investigated in trials, most comparative studies focus on acupuncture vs Western medications or placebo rather than direct comparisons between different acupuncture modalities. This creates an evidence gap for clinicians selecting optimal acupuncture protocols. The inherent diversity of acupuncture interventions poses challenges for conventional meta-analyses and systematic reviews in addressing these clinical questions, highlighting the need for more head-to-head comparative studies among acupuncture techniques. Network meta-analysis (NMA) can help address this dilemma by comparing multiple interventions when there is insufficient direct evidence to compare all therapies. By combining both direct comparisons from randomized controlled trials (RCTs) and indirect comparisons through a common comparator, NMA allows the simultaneous estimation of relative treatment effects among multiple competing interventions within a single analytical framework. This approach has been increasingly adopted to provide a coherent synthesis of evidence and to facilitate the comparative evaluation and ranking of treatment options in complex clinical decision-making contexts [20]. Its analytical framework extends beyond the scope of pairwise head-to-head comparative meta-analyses [21]. By systematically comparing multiple competing interventions across different but sufficiently comparable study populations and clinical contexts, this approach enables the evaluation of broader clinical questions within an expanded evidence synthesis framework [22].

In this study, an NMA protocol is designed to determine the most efficacious acupuncture interventions (individual or combined therapies) for managing CINV in patients with breast cancer, using evidence derived from RCTs. Additionally, their safety profiles and subgroup therapeutic effects will be systematically evaluated.

## Methods

This NMA protocol rigorously follows the PRISMA-P (Preferred Reporting Items for Systematic Reviews and Meta-Analyses Protocols) 2015 statement (Checklist 1) [23,24]. This protocol is registered in the PROSPERO database (CRD420251030300).

### Ethical Considerations

Ethics approval is not required as this study is based on publicly available data and does not involve animal testing.

### Eligibility Criteria

The literature screening process is conducted according to the population, intervention, comparison, outcome, and study (PICOS) framework.

#### Population

Eligible participants include adult female patients (aged ≥18 years) with a pathologically confirmed diagnosis of breast cancer who are undergoing moderately or highly emetogenic chemotherapy and consent to receive acupuncture-related interventions. Patients receiving concurrent radiotherapy or with pre-existing gastrointestinal comorbidities, immunodeficiencies, or physical or cognitive impairments will be excluded. No restrictions are applied regarding nationality, ethnicity, education level, marital status, or socioeconomic background.

#### Interventions

Considering the heterogeneity of TCM acupuncture-related therapies, this review will exclusively include modalities frequently used in CINV management. These include hand needling, electroacupuncture, auricular acupressure, fire needling, warm needling, catgut embedding, intradermal needling, abdominal acupuncture, acupressure, or a combination of these methods. The interventions of plum-blossom needling, dry needling, wrist-ankle acupuncture, acupoint patching, acupoint injection, and moxibustion are outside the scope of this study. We do not set limitations on the specific acupoint selection or treatment duration.

#### Comparators

Control interventions include placebo acupuncture, sham acupuncture, conventional antiemetic pharmacotherapy (eg, various 5-HT3 RAs), and other acupuncture methods as described previously. Studies investigating variations within the same acupuncture technique, such as comparisons between alternative acupoint choices or different treatment durations, are systematically eliminated.

#### Outcomes

##### Primary Outcomes

CINV intensity and clinical effectiveness rates are the primary outcomes of this review.

CINV intensity (continuous outcome) may be quantified using multiple validated assessment tools [16,25-27] (eg, visual analog scale, Multinational Association of Supportive Care in Cancer Antiemetic Tool, Simulator Sickness Questionnaire, Rhodes Index of Nausea, Vomiting and Retching, and the Index of Nausea, Vomiting, and Retching). To enable quantitative synthesis across studies using different instruments, we will treat CINV intensity as a continuous outcome and standardize it using a common effect size. If a study reports multiple time points, we will extract the time point closest to the end of the intervention.

Clinical effectiveness (dichotomous outcomes) is assessed based on the World Health Organization (WHO) criteria for grading CINV and established acupuncture efficacy standards. Clinical efficacy is categorized into 4 levels [28]: complete response (CR; no nausea or vomiting), partial response (PR; mild nausea not affecting appetite and vomiting 1 to 2 times per day), mild response (moderate nausea impairing appetite and vomiting 3 to 5 times per day), and failure (severe nausea with >5 episodes of vomiting per day). The overall effectiveness rate is computed as follows: [(number of patients with CR+PR)/total number of patients]×100%. Additionally, CR has been set as an outcome indicator in some studies. While the National Cancer Institute Common Toxicity Criteria exhibits minor classification discrepancies compared with WHO standards, their inclusion is permitted due to negligible impact on efficacy calculations [29,30]. Studies lacking explicit definitions of CR or PR will be excluded to ensure outcome reliability.

##### Secondary Outcomes

Secondary outcomes include CINV recurrence rates after the intervention; patients’ quality of life, evaluated using related tools (eg, Quality of Life questionnaire-core 30 and Beck Anxiety Inventory); and safety profiles covering the type and incidence of adverse events.

### Studies

Only parallel-designed RCTs are included, while crossover trials, case-control studies, cohort studies, and animal or cellular tests are not considered. The language of the studies is confined to English or Chinese. We also excluded conference abstracts, case reports, reviews, guidelines, letters, and studies with missing content.

### Search Strategy

A thorough electronic search of 8 major biomedical databases (PubMed, Embase, Web of Science, the Cochrane Library, China National Knowledge Infrastructure, Wanfang Database, VIP Database, and the Chinese Biomedical Literature Database) will be implemented from their respective inception dates to July 31, 2025. The search terms will consist of standardized Medical Subject Headings (MeSH), TCM-related terms, and free keywords. As an illustration, the PubMed search strategy is outlined in Multimedia Appendix 1, with full details provided therein. The search formula will be subject to targeted minor modifications according to the search requirements for different databases. Non-English or non-Chinese records will be excluded during screening, and such exclusions will be documented in the PRISMA (Preferred Reporting Items for Systematic reviews and Meta-Analyses) flow diagram.

### Study Selection

**Figure 1.**
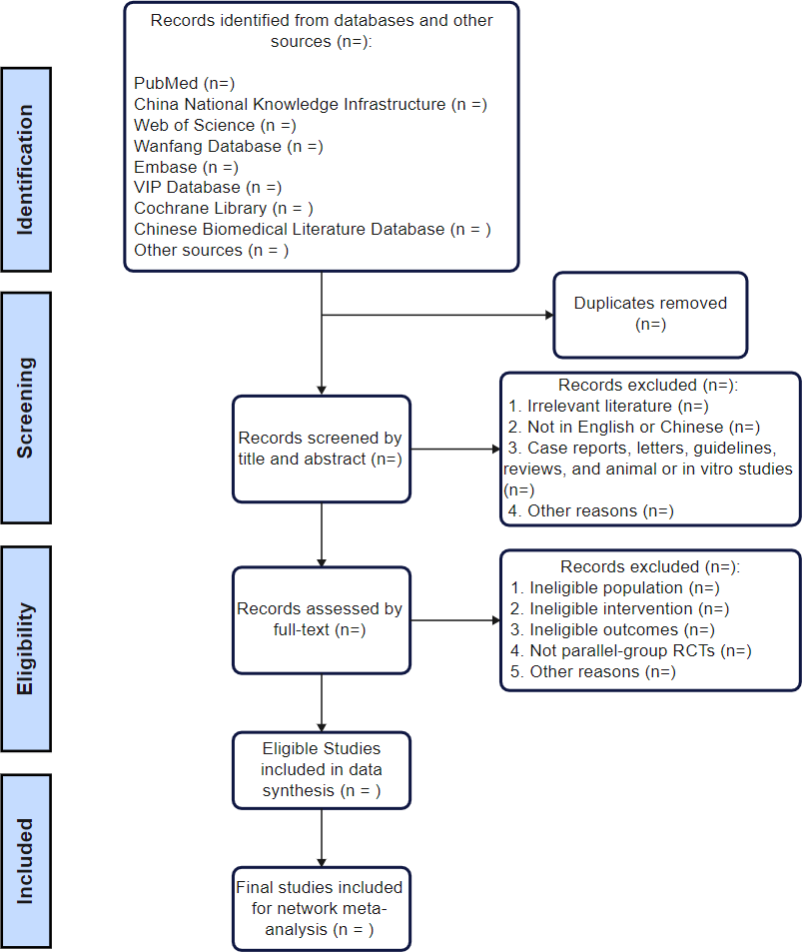
The process for records selection. RCT: randomized controlled trial.

Study selection is independently conducted by 2 reviewers (YL and ZB), who will screen all retrieved studies according to the eligibility criteria. All conflicts in selection are resolved by a third reviewer (HZ), who specializes in diagnosing and treating breast cancer. The search and screening process is depicted in Figure 1. All retrieved research will be imported into EndNote (version X8.2; Clarivate). Initially, duplicate literature will be eliminated using this software. After that, the titles and abstracts of the records will be read for preliminary filtering to remove irrelevant and ineligible studies, followed by a thorough review of the remaining studies to select the final included studies. If the data are incomplete in a study, we will contact the corresponding author to request additional details; the study will be excluded if the author cannot be reached or the missing information cannot be obtained.

### Data Extraction

Two researchers (YL and TL) will systematically extract data from eligible studies into a standardized Microsoft Excel 2019 template, capturing the following elements: literature details (authors, publication year, journal, and country), trial characteristics (blinding methods, randomization processes, number of participants, and chemotherapy scheme), intervention (acupuncture method and acupoint selection), comparator (control measures and Western medicine names), and outcome (preintervention or postintervention values, outcome criteria, and their authoritative sources). Particular attention is given to distinguishing chemotherapy cycle phases and recording acute CINV (≤24 hours) and delayed CINV (24 hours to 7 days) separately. To minimize extraction bias, standardized conversion methods will be applied for studies reporting data in unconventional formats. For continuous outcomes, means and SDs will be estimated from medians, ranges, or IQRs using validated formulas. SEs and CIs will also be converted according to the Cochrane Handbook. For dichotomous outcomes, if only event counts are reported, the corresponding percentages will be calculated to ensure data consistency. For 3-armed trials, comparisons between the intervention and control groups will be appropriately structured—such as by splitting the shared control group—to enable inclusion as pairwise comparisons in the analysis. If disagreements arise, a third reviewer (HZ) will be invited to reach a consensus when necessary.

### Risk of Bias Assessment

The Cochrane risk of bias tool [31] will be used to evaluate the quality of the included literature, which mainly includes 6 aspects of the risk of bias. Selection bias mainly evaluates the randomness of the grouping method (eg, random number table and computer-generated sequences) and the allocation concealment (eg, sealed envelope methods). Blinding during implementation and blinding of outcome assessment are considered performance bias and detection bias, respectively, and are categorized as single-blind, double-blind, or unblinded. Attrition bias focuses on study participant attrition and aims to evaluate whether participant absences and their reasons are truthfully recorded. Reporting bias is assessed to ensure the authenticity and integrity of the study data. Any additional elements that could cause bias are categorized as other biases. All studies are evaluated by 2 independent assessors, and each bias will be categorized into 3 grades: low risk (meets the criteria), high risk (criteria deviant), and unclear risk (inadequate evidence to make a judgment), with adjudication by a third reviewer if disagreements persist. The results will be presented as a bias risk diagram by the Review Manager (Cochrane Collaboration).

### Statistical Analysis

#### Data Integration

Initially, pairwise meta-analysis will be carried out to directly compare the effect of various acupuncture modalities for CINV. Given potential clinical and methodological variations across studies, pairwise syntheses will primarily use random-effects models. For CINV intensity measured by different instruments, we will use the standardized mean difference with Hedges *g* small-sample correction as the common comparative effect size and report the 95% CIs. All intensity scales will be oriented such that negative values represent symptom improvement. For clinical effectiveness outcomes, we will use the risk ratio as the effect size with 95% CIs. The 95% CIs will be computed using the Hartung-Knapp-Sidik-Jonkman method to enhance inference reliability and reduce inflated type I error rates, particularly when the number of studies is small [32]. These steps will be performed using Stata (version 17.0; StataCorp).

A Bayesian probabilistic NMA will be implemented in R (version 4.4.1; R Foundation for Statistical Computing) “*gemtc*” package, if the transitivity assumption is valid. Separate NMAs will be performed for continuous and dichotomous outcomes, specifying appropriate likelihoods and link functions for each outcome type (normal likelihood with an identity link for standardized mean difference; binomial likelihood with a log link for risk ratio). We will conduct data analysis with the Markov chain Monte Carlo model and the random-effects model. Four independent chains will be set up, and 50,000 iterations will be run for each chain. Visual assessment of the convergence will be performed using Gelman-Rubin statistics and trace plots [33,34]. An R-hat value approaching 1 indicates good convergence, trace plots should exhibit stable fluctuations, and autocorrelation should be low. To offer reliable data support for clinical decisions, we will rank the efficacy of varying acupuncture methods. Treatment ranking will be demonstrated by the surface under the cumulative ranking curve; the larger the surface under the curve, the greater the acupuncture therapy effectiveness.

#### Network Geometry

Network graphs will be plotted to demonstrate the evidence relationships between interventions. Given that separate NMAs will be conducted for different primary outcomes, network plots will be generated separately for each outcome type using studies that report the corresponding outcome. In the graphs, nodes will represent each treatment modality (eligible acupuncture treatments) and control interventions (sham acupuncture, placebo needling, and conventional drugs), with their diameters proportional to the total sample size of the corresponding interventions. The baseline will be set at 6 mm for 200 patients, with an increase of 1 mm for each additional 50 patients. The connecting lines between nodes indicate direct comparative evidence, and the thickness of lines is positively affected by the number of comparative studies. One study will be set to a line thickness of 1 point, with each additional 2 studies increasing the thickness by 0.3 point, up to a maximum width of 2.5 points to prevent graphical distortion. The absence of a line denotes missing head-to-head comparisons. We will assess network density by calculating the ratio of actual direct comparisons to the theoretical maximum possible number to quantify network completeness.

#### Heterogeneity

The *Q* test and *I*^2^ statistics will be used to evaluate heterogeneity across the included studies. Acknowledging the limitations of *I*^2^, we will emphasize the between-study variance (τ^2^) and its square root (τ) to quantify the extent to which true effects vary across studies. Furthermore, to assess the practical implications of this variation in real-world clinical settings, 95% prediction intervals will be calculated when at least 10 studies are available for a given comparison, thereby complementing the 95% CI for the pooled average effect [35]. In cases where substantial heterogeneity is detected, we will perform subgroup analyses and meta-regression to identify possible contributing factors [36]. Furthermore, node-splitting forest plots will be generated using Stata to visualize variation among direct and indirect evidence for each intervention pair.

#### Consistency

Consistency analyses enhance the credibility of clinical efficacy rankings. The following strategy will be implemented to ensure consistency among direct and indirect evidence. The node-splitting method will be applied to calculate discrepancies between direct and indirect evidence for each pair of interventions [37]. Local inconsistency will be claimed if the node-splitting *P* value is <.10. Moreover, global network consistency will be estimated using the design-by-treatment interaction model [38], with the consistency assumption accepted if the global *P* value is >.10. All analyses will follow a frequentist framework and report inconsistency factors with 95% CIs.

#### Subgroup Analysis and Meta-Regression

The following variables will be considered in the subgroup analyses: participant characteristics (eg, chemotherapy regimens, cycle sequence, and stage of breast cancer), outcome measures (eg, scale type and clinical treatment rate [effectiveness rate vs CR rate]), and study region (China vs Europe and America).

Covariates involved in the meta-regression will include study sample size, average age of patients, number of acupuncture treatments, and others.

#### Small-Study Effects and Publication Bias

In complex intervention networks, small-study effects and publication bias may lead to distorted efficacy rankings. We will assess potential small-study effects using comparison-adjusted funnel plots and statistical tests. When at least 10 studies are included, effect sizes will be calculated and comparison-adjusted funnel plots will be generated using Stata [39]. Funnel plot asymmetry will be interpreted as an indication of potential small-study effects rather than definitive evidence of publication bias, as publication bias is only one of several possible explanations, including clinical heterogeneity and methodological limitations [40,41]. To further investigate these effects, the Egger test will be performed. If small-study effects are detected, we will conduct sensitivity analyses to evaluate the robustness of our findings [42].

#### Quality of Evidence

The rigor of included evidence will be evaluated using the Grading of Recommendations, Assessment, Development, and Evaluation (GRADE) framework [43]. We will systematically evaluate 5 critical areas (risk of bias, indirectness, inconsistency, imprecision, and publication bias) through the GRADEpro GDT (Evidence Prime Inc) [44]. Each study will be classified into 4 grades of quality: high, moderate, low, or very low. Any discrepancies in grading will be settled through iterative discussions until mutual agreement is achieved. The final evidence profiles will be synthesized into summary of findings tables, explicitly documenting downgrading rationales for each assessed domain to ensure transparency in quality judgments. The final evidence will be reported in a format that specifies the reasons for downgrading in each area to ensure the reliability of the quality assessment.

## Results

A preliminary scoping search was concluded in August 2025 to refine the research question. This protocol was finalized in October 2025. The comprehensive literature search and study selection are expected to be completed by June 2026, followed by data extraction by August 2026. Data synthesis and final manuscript preparation are scheduled to be completed by December 2026. The results will be disseminated through open-access publications.

## Discussion

### Overview

As one of the most prevalent malignancies globally, breast cancer requires focused attention on the management of chemotherapy-related side effects, particularly CINV, a common complication that significantly affects quality of life. Acupuncture’s therapeutic value has been extensively demonstrated in international clinical practice and has gained increasing patient acceptance. A growing number of clinicians, particularly TCM physicians and acupuncturists, have focused on this specialized therapy for CINV in patients with breast cancer.

We note that several published meta-analyses have investigated acupuncture for CINV in breast cancer. Issac et al [45] demonstrated the superiority of acupressure in controlling acute nausea, delayed nausea, and delayed vomiting. Jang et al [46] examined acupuncture’s effects on a variety of chemotherapy-related complications in breast cancer, with 2 trials addressing nausea and vomiting management. However, the limitation of these 2 studies lies in their narrow focus on the efficacy of isolated acupoint therapies. The analysis by Wang et al [47] broadly compared nonpharmacological interventions for CINV in all tumors with some acupuncture modalities. In contrast, our study exclusively evaluates multiple acupuncture therapies in patients with breast cancer and incorporates more comprehensive outcomes. This deliberate exclusion of herb-assisted techniques (eg, acupoint patch) eliminates potential confounding from pharmaceutical effects, allowing for a clearer interpretation of traditional acupuncture’s intrinsic efficacy in this specific population.

While various acupuncture methods have been widely implemented in China for managing CINV in patients with breast cancer, international clinical practice, especially in Europe and the United States, remains dominated by electroacupuncture, auricular acupuncture, and acupressure, with limited use of some techniques such as fire needling, abdominal acupuncture, and intradermal needling. Emerging evidence from Chinese clinical trials has demonstrated the effects of these techniques [48,49].

### Strengths and Limitations

The primary strength of our research lies in the systematic comparison of multiple acupuncture modalities for mitigating CINV in patients with breast cancer using NMA, with the aim of providing clinicians with a broader range of evidence-based therapeutic selection. This protocol might also facilitate the use of acupuncture and moxibustion for CINV in other malignancies or for other side effects generated by chemotherapy.

Some potential limitations and difficulties may arise from the conduct of this research. First, the 9 acupuncture modalities included exhibit clinical variability (eg, differences in stimulation intensity), compounded by diverging outcome assessment criteria between Chinese and Western studies, which may cause significant heterogeneity. Second, despite using unrestricted geographic or ethnic search strategies, we observed that the literature search reveals a predominance of Asian studies, likely attributable to regional variations in acupuncture acceptance. This phenomenon will raise concerns about potential biases stemming from ethnicity-related genetic and cultural factors. Finally, nowadays, most RCTs tend to adopt the design of “acupuncture combined drug vs drug” [50,51], which may create insufficient direct evidence comparing acupuncture monotherapy with pharmacological interventions and limit assessment of acupuncture’s independent efficacy. Future trials are suggested to adopt 3-arm designs (acupuncture alone, drug alone, and combination therapy) to clarify absolute therapeutic benefits.

### Conclusions

This protocol outlines the methodology for a systematic review and NMA that will comprehensively compare the efficacy of multiple acupuncture modalities for managing CINV in patients with breast cancer. By integrating both conventional Western and TCM outcome indicators, this study is positioned to generate nuanced, clinically relevant evidence. The findings will not only expand the range of evidence-based nonpharmacological options available to clinicians but also facilitate the application of acupuncture in supportive oncology care.

## Supplementary material

10.2196/86384 Multimedia Appendix 1 PubMed search strategy.

10.2196/86384 Checklist 1 PRISMA-P 2015 checklist.

## Abbreviations

5-HT: 5-hydroxytryptamine
CINV: chemotherapy-induced nausea and vomiting
CR: complete response
GRADE: Grading of Recommendations, Assessment, Development, and Evaluation
MeSH: Medical Subject Headings
NK-1: neurokinin-1
NMA: network meta-analysis
PICOS: population, intervention, comparison, outcome, and study
PR: partial response
PRISMA: Preferred Reporting Items for Systematic reviews and Meta-Analyses
PRISMA-P: Preferred Reporting Items for Systematic Reviews and Meta-Analyses Protocols
RA: receptor antagonist
RCT: randomized controlled trial
TCM: traditional Chinese medicine
WHO: World Health Organization
