# Supplementary material for: Acupuncture Therapies for Chemotherapy-Induced Nausea and Vomiting in Patients With Breast Cancer: Protocol for a Systematic Review and Network Meta-Analysis
Source: JMIR Res Protoc. 2026 Apr 21;15:e86384. doi: 10.2196/86384 (PMC13099031; doi:10.2196/86384)
Supplement: Multimedia Appendix 1 [file resprot-v15-e86384-s001.docx]

Multimedia Appendix 1. Search Strategy of PubMed

| No. | Search Terms |
| --- | --- |
| #1 | breast neoplasm[Mesh] OR "breast neoplasm*"[Ti/Ab] OR "breast cancer"[Ti/Ab] OR "breast tumor*"[Ti/Ab] OR "breast carcinoma*"[Ti/Ab] OR "cancer of breast"[Ti/Ab] OR "cancer of the breast"[Ti/Ab] OR "breast malignant neoplasm*"[Ti/Ab] OR "Mammary Carcinoma*"[Ti/Ab] OR "mammary neoplasm*"[Ti/Ab] OR "mammary cancer*[Ti/Ab] OR "breast malignant tumor*"[Ti/Ab] OR "malignant neoplasm of breast"[Ti/Ab] OR "malignant tumor of breast"[Ti/Ab] |
| #2 | "acupuncture" [Mesh] OR "acupuncture therapy" [Mesh] OR "Acupuncture, Ear"[Mesh] OR "acupressure"[Mesh] OR "acupuncture*"[Ti/Ab] OR "moxibustion*"[Ti/Ab] OR "acupressure" [Ti/Ab] OR "electroacupuncture" [Ti/Ab] OR "auricular acupressure"[Ti/Ab] OR "auriculotherapy"[Ti/Ab] OR "acupoint*"[Ti/Ab] OR "acupoint injection*"[Ti/Ab] OR "acupoint catgut embedding"[Ti/Ab] OR "needle"[Ti/Ab] OR "warm needle"[Ti/Ab] OR "pharmacoacupuncture*"[Ti/Ab] OR "acupotomy*"[Ti/Ab] OR "traditional Chinese medicine"[Ti/Ab] OR "TCM"[Ti/Ab] OR "traditional medicine"[Ti/Ab] |
| #3 | "chemotherapy-induced nausea and vomiting"[Ti/Ab] OR "CINV"[Ti/Ab] OR "nausea and vomiting"[Ti/Ab] OR "emesis*"[Ti/Ab] OR "nausea*"[Ti/Ab] OR "vomiting*"[Ti/Ab] |
| #4 | "randomized controlled trial"[Publication Type] OR "randomly" [Ti/Ab] OR "randomized"[Ti/Ab] OR "randomised"[Ti/Ab] OR "trial"[Ti/Ab] OR "controlled clinical trial"[Publication Type] |
| #5 | #1 AND #2 AND #3 AND #4 |

Ti: Title, Ab: Abstract
