# Supplementary material for: Acupuncture Therapies for Chemotherapy-Induced Nausea and Vomiting in Patients With Breast Cancer: Protocol for a Systematic Review and Network Meta-Analysis
Source: JMIR Res Protoc. 2026 Apr 21;15:e86384. doi: 10.2196/86384 (PMC13099031; doi:10.2196/86384)
Supplement: Checklist 1 [file resprot-v15-e86384-s002.pdf]

Research Checklist - PRISMA-P (Preferred Reporting Items for Systematic review and Meta-Analysis Protocols) 2015 checklist

Section/topic	#	Checklist item	Information reported		Line number(s)
			Yes	No	
**ADMINISTRATIVE INFORMATION**					
**Title**					
Identification	1a	Identify the report as a protocol of a systematic review	☒	☐	***Page1/line1-3***
Update	1b	If the protocol is for an update of a previous systematic review, identify as such	☐	☒	***Not applicable***
**Registration**	2	If registered, provide the name of the registry (e.g., PROSPERO) and registration number in the Abstract	☒	☐	***Page2/line52***
**Authors**					
Contact	3a	Provide name, institutional affiliation, and e-mail address of all protocol authors; provide physical mailing address of corresponding author	☒	☐	***Page1/line5-15***
Contributions	3b	Describe contributions of protocol authors and identify the guarantor of the review	☒	☐	***Page14/line439-444***
**Amendments**	4	If the protocol represents an amendment of a previously completed or published protocol, identify as such and list changes; otherwise, state plan for documenting important protocol amendments	☐	☒	***Not applicable***
**Support**					
Sources	5a	Indicate sources of financial or other support for the review	☒	☐	***Page13/line426-430***
Sponsor	5b	Provide name for the review funder and/or sponsor	☒	☐	***Page12/line426-430***
Role of sponsor/funder	5c	Describe roles of funder(s), sponsor(s), and/or institution(s), if any, in developing the protocol	☐	☒	***None***
**INTRODUCTION**					
**Rationale**	6	Describe the rationale for the review in the context of what is already known	☒	☐	***Page2-5/line55-139***
**Objectives**	7	Provide an explicit statement of the question(s) the review will address with reference to participants, interventions, comparators, and outcomes (PICO)	☒	☐	***Page5/line140-144***

Section/topic	#	Checklist item	Information reported Yes	Information reported No	Line number(s)
**METHODS**					
**Eligibility criteria**	8	Specify the study characteristics (e.g., PICO, study design, setting, time frame) and report characteristics (e.g., years considered, language, publication status) to be used as criteria for eligibility for the review	☒	☐	*Page5-7/ line151-207*
**Information sources**	9	Describe all intended information sources (e.g., electronic databases, contact with study authors, trial registers, or other grey literature sources) with planned dates of coverage	☒	☐	*Page7/line209-212*
**Search strategy**	10	Present draft of search strategy to be used for at least one electronic database, including planned limits, such that it could be repeated	☒	☐	*Figure 1*
STUDY RECORDS					
Data management	11a	Describe the mechanism(s) that will be used to manage records and data throughout the review	☒	☐	*Page7/line224，234*
Selection process	11b	State the process that will be used for selecting studies (e.g., two independent reviewers) through each phase of the review (i.e., screening, eligibility, and inclusion in meta-analysis)	☒	☐	*Page7/line220-230*
Data collection process	11c	Describe planned method of extracting data from reports (e.g., piloting forms, done independently, in duplicate), any processes for obtaining and confirming data from investigators	☒	☐	*Page7-8/ line233-250*
**Data items**	12	List and define all variables for which data will be sought (e.g., PICO items, funding sources), any pre-planned data assumptions and simplifications	☒	☐	*Page7-8/ line234-239*
**Outcomes and prioritization**	13	List and define all outcomes for which data will be sought, including prioritization of main and additional outcomes, with rationale	☒	☐	*Page6/ line176-202*
**Risk of bias in individual studies**	14	Describe anticipated methods for assessing risk of bias of individual studies, including whether this will be done at the outcome or study level, or both; state how this information will be used in data synthesis	☒	☐	*Page8/ line252-266*
DATA					
**Synthesis**	15a	Describe criteria under which study data will be quantitatively synthesized	☒	☐	*Page9/line272-279*
	15b	If data are appropriate for quantitative synthesis, describe planned summary measures, methods of handling data, and methods of combining data from studies, including any planned exploration of consistency (e.g., I^2, Kendall's tau)	☒	☐	*Page8/line241-249; Page8-9 /line269-292; Page10/line310-320*

Section/topic	#	Checklist item	Information reported		Line number(s)
			Yes	No	
	15c	Describe any proposed additional analyses (e.g., sensitivity or subgroup analyses, meta-regression)	☒	☐	***Page10/line322-338***
	15d	If quantitative synthesis is not appropriate, describe the type of summary planned	☐	☒	***Not applicable***
**Meta-bias(es)**	16	Specify any planned assessment of meta-bias(es) (e.g., publication bias across studies, selective reporting within studies)	☒	☐	***Page10-11/ line340-349***
**Confidence in cumulative evidence**	17	Describe how the strength of the body of evidence will be assessed (e.g., GRADE)	☒	☐	***Page11/line351-361***
